# Epstein barr virus encodes miRNAs to assist host immune escape

**DOI:** 10.7150/jca.42498

**Published:** 2020-02-03

**Authors:** Weiming Li, Cong He, Jiayi Wu, Dazhi Yang, Weihong Yi

**Affiliations:** 1 Department of orthopedics, Union Shenzhen Hospital, Huazhong University of Science and Technology, Shenzhen, Guangdong, China; 2 Department of orthopedics, The 6th Affiliated Hospital of Shenzhen University Health Science Center, Shenzhen, Guangdong, China

**Keywords:** EBV miRNAs, immune escape, tumor

## Abstract

Epstein-barr virus (EBV) is a definite tumorigenic virus, which can form life-long latency in the host, which is difficult to be recognized and completely eliminated by the immune system. It is closely related to the occurrence and development of nasopharyngeal cancer, gastric cancer and various types of lymphoma. At present, a total of 44 Epstein-barr virus-encoded microRNAs (EBV miRNAs) have been found. In response to the immune system of the body, EBV miRNAs can inhibit the expression and presentation of viral antigens, inhibit immune activation and immunotoxicity, assisting host cells to escape from immunity, and providing conditions for further immortalized tumorigenesis of the host cells.

## Introduction

EBV is a human herpesvirus type IV, belonging to the human herpesvirus gamma subfamily 1. It was first discovered and named by Anthony Epstein and Yvonne Barr in 1964 2. EBV is a harmful pathogen and is closely related to the occurrence of nasopharyngeal carcinoma and Burkitt's lymphoma. More and more evidence indicates that EBV is also the cause of Hodgkin's lymphoma, B and T/NK non Hodgkin lymphomas and gastric cancer 3-6. Currently, it is known that EBV has caused at least 200,000 new cancer patients every year 7.

EBV infection is mainly transmitted through saliva, but can also be infected through blood transfusion, and the infection rate in adults is as high as 90% 8. The first infection of EBV occurs mainly in the epithelial cells of the pharynx of the population, followed by infection of B lymphocytes. As B lymphocytes carrying EBV enter the blood circulatory system, systemic EBV infection can occur 9, 10. In the face of host immune response and immunotoxicity, EBV can derive corresponding strategies to assist the host cells to immune escape, and establish a lifetime of latency, which is difficult to be completely eliminated by the body's immune system 11.

After EBV infects the host, EBV antigen is encoded and expressed. Currently, specific antigens including Epstein-barr virus nuclear antigen (EBNA), Epstein-barr virus capsid antigen (EBVCA), Epstein-barr virus early antigen (EA), Epatein-barr virus membrane antigen (MA), etc. 12. EBNA is a latent gene of EBV, in addition to latent genes such as EBERs, BARTs, and EBNA-LP. According to the latent genes expressed by EBV in host cells, hosts can be classified into Four latency (type 0, type I, type II, and type III) 13, 14. The EBV of type 0 latency expresses only one latent gene in the host cell is EBV-encoded RNAs (EBERs). For example, infected dormant memory B cells belong to type 0 latency 15. The EBV of type I latency expresses three latent genes in the host cell, EBNA1, EBERs, and BARTs (BamHI-A rightward transcripts). Such as burkitt's lymphoma and plasmablastic lymphoma, which belong to type I 16, 17. The latent genes of EBV expression in the host with type II latency include four latent genes: EBNA1, EBERs, BARTs, LPMs (latent membrane protein). For example, hodgkin's lymphoma, nasopharyngeal carcinoma, diffuse large B-cell lymphoma, chronic lymphocytic leukemia and EBV-associated gastric cancer are type II latency 18-21. The hosts with type III latency express eight latent genes, such as EBERs, EBNA1, EBNA-LP (EBNA-leader protein), EBNA2, EBNA3A, EBNA3B, EBNA3C, LPMs. For example, immunoblastic lymphoma, diffuse large B of the elderly and pyothorax associated lymphoma belong to type III 19, 22.

Interestingly, EBV is usually a type I latency in PBL patients, expressing EBNA1, EBERs, and BARTs 16, 23. However, in a recent study of miRNA sequencing in PBL, it was found that EBV also had type II latency in PBL patients and expressed LMP1, suggesting that the latency state of EBV in tumor is not constant, and the change of latency may be related to immune regulation of EBV^24^. PBL patients are often associated with HIV and EBV infection 25, 26. HIV infection has long been considered as the main cause of PBL, while EBV infection has not received much attention. Rodrigues-Fernandes, C.I., et al. made a clinicopathological analysis of 159 cases of PBL, and found that EBV positive status was one of the independent prognostic determinants of a poor prognosis through a multivariate Cox regression model 27. In addition, Gravelle, P and Laurent, C, et al. found that compared with EBV negative PBL, there were abundant leukocyte infiltration and T cell activation signals in EBV positive PBL tissue, high expression of PD-1/PD-L1 immune checkpoint and other immune escape related proteins, and positive correlation with host immunosuppression, suggesting EBV may play a more important role in immune escape 16, 28. In the study of miRNA sequencing in PBL also found that the expression of tumor suppressor gene PTEN, PBX2 and metabolic controller PPARGC1A, PLIN2/adipophilin was regulated by BART-19-5p 24. These studies suggest that EBV plays an important immunomodulatory role in the development of PBL, during which the latency status of EBV may change, and EBV-miRNAs may be involved in different regulatory roles.

EBV not only has different types of latency in different hosts and different expressed latent genes but also has different expressions of miRNAs encoding itself in different host tissues with types of latency. For example, EBV-miR-BARTs are widely expressed in the four latency types and lytic replication of EBV in host cells, while the expression of EBV-miR-BHRF1s depends on the type of virus latency and is generally expressed in III latency and lytic replication of host cells 29, 30. In addition, the expression of EBV-miR-BHRF1s in lymphoma is very high, while the expression in nasopharyngeal and epithelial tumors is absent 19. EBV-miR-BARTs are expressed in both lymphoma and epithelial tumors. These studies suggest that EBV-miR-BARTs and EBV-miR-BHRH1s play different regulatory roles in different tumors and different latency.

## EBV miRNAs

EBV is the first virus discovered by humans to express miRNAs. EBV miRNAs are non-coding small RNA composed of 18-24 nucleotides. So far, a total of 25 EBV miRNAs precursors have been found, forming 44 mature EBV-miRNAs, which are transcribed from the two precursors to the two regions of the EBV genome, namely the BART region and the BHRF1 region, wherein the BART region has two miRNA cluster regions (BART-Cluster 1, BART-Cluster 2) and the separate BART2 region, can transcribe 22 miRNAs precursors to form 40 mature miRNAs. The miRNAs BART-Cluster 1 is located between the BART gene exon 1 and exon 1B and encodes 8 miRNA precursors (EBV-miR-BART1, EBV-miR-BART3~6, and EBV-miR-BART15~17). BART-Cluster 2 is located between exon 1B and exon 3 of the BART gene and encodes 13 miRNA precursors (EBV-miR-BART7~14 and EBV-miR-BART18~22). While the BART2 region is located between exon 4 and exon 5, encodes only one precursor (EBV-miR-BART2). The BHRF1-Cluster region transcribes three miRNA precursors (EBV-miR-BHRF1-1~3) to form four mature EBV-miR-BHRF1s. EBV miRNAs are named EBV-miR-BART1~22 and EBV-miR-BHRF1-1~3 according to the transcription region of their genome and the order in which they were found 31-33. Interestingly, not all EBV strains have complete BART region. The B95-8 laboratory strain of EBV bears a deletion that removes most of the BART region, can immortalize primary B cells in culture, suggesting that BARTs are dispensable for transforming B cells 34.

In a typical miRNA organism, one chain is selective processing into mature miRNA, while the other strand is rapidly degraded, but EBV miRNAs during processing are not all so, most of the EBV miRNAs hairpin structure function related to the two chains are processed into mature miRNA, to distinguish the two miRNAs, according to the 3' or 5' end of the miRNA in the hairpin structure named suffix -3p or -5p 35, 36. Therefore, 25 precursors eventually form 44 mature EBV miRNAs.

After EBV infection of host cells, these EBV miRNAs form incomplete complementary pairing with the 3'-untranslated region (3'UTR) of the virus's own genes or host gene mRNAs, resulting in inhibition of gene translation or the degradation of targeted mRNAs 37. In recent years, many studies have shown that EBV miRNAs can inhibit the expression and presentation of viral antigens by inhibiting the expression of viral genes and host cell genes, inhibiting the activation and cytotoxicity of immune cells, and achieving long-term latency, immune escape, and promotion of host cells immortalization and tumor development 38, which is briefly reviewed below.

## Antigen expression

EBV infected host cell for the first time, to remove the EBV infection, host cells will present the EBV antigens to the cell surface, so that through cell immune clearance of the virus in the cell. In order to evade the body's immune system to kill, EBV miRNAs can regulate host cell EBV antigen expression, to present a lower amount of antigen, and difficult to be immune cells to recognize, to escape immune surveillance at an early stage, create conditions for the latency of EBV 39. For example, LMP2A is EBV encoded with high immunogenicity of virus antigen, which can be CD4+ and CD8+ T cells specificity recognition 40. But Lung, R.W., et al. found that in EBV+ nasopharyngeal cells, EBV-miR-BART22 targets the 3'UTR of LMP2A gene mRNA, inhibits the expression of LMP2A, weakens the host's immune response, and makes EBV+ nasopharyngeal cells become a blind area for immune surveillance and immune escape 41.

LMP1, as an EBV-encoded viral antigen, is a kind of oncogenic gene and participates in the activation of signaling pathways such as NF-κB, JNK-p38/SAPK, Ras-MAPK, PI3k-Akt and JAK-STAT, which can promote the occurrence of EMT and make nasopharyngeal carcinoma has a higher invasion and metastasis ability, which is considered to be an important factor in the development of nasopharyngeal carcinoma 42. Studies have found that EBV-miR-BART9, EBV-miR-BART16 and EBV-miR-BART17-5p can both target the 3'UTR of LMP1 mRNA and negatively regulate the expression of LMP1. Although such negative regulation of LMP1 can reduce the invasion and metastasis of tumor cells to a certain extent, it also greatly weakens the host's immune recognition and response, and evade immune surveillance 43-45. And Lo, A.K., et al. found that very small amounts of LMP1 (below the detection threshold), enough to activate the NF-κB signaling pathways, play a role in promoting the cancer. While this small amount of LMP1, compared with the sensitivity of memory T cells to the LMP1 epitope, cannot activate memory T cells of the immune system. This suggests that EBV miRNAs may, by fine-regulating the expression level of LMP1, promote host immortalization of tumors while avoiding immune surveillance and promote the development of tumors 46.

EBNA1 is the first discovered EBV antigen, expressed in host cells of various latency types at the proliferative stage. Although EBNA1 can be T cells target recognition, the host of EBNA1 almost not be MCH I presented to the surface of cells. Studies also found that EBV miRNAs can inhibit EBNA1 expression, which may be EBV miRNAs a means to assist the host to escape immune surveillance 47. In addition, EBV miR-BART6-5p was found to inhibit EBNA2 expression 48, and EBV-miR-BHRF1s could inhibit EBNA2 co-activated antigen EBNA-LP expression 49, suggesting that EBNAs and EBNA-LP could be regulated by EBV miRNAs to avoid immune surveillance and maintain EBV latency.

## Antigen presentation

After EBV infection of the host, to avoid exposure by host cells, evade immune system recognition, EBV miRNA not only participates in the regulation of EBV antigen expression but also regulates the presentation of EBV antigen during the process of EBV antigen transport to the host surface. It can avoid large amounts of antigen exposure and be recognized and attacked by immune cells, helping the host immune escape. After EBV infection, the antigen peptide segment formed by EBV antigen in the cytoplasm after treatment by proteasome enzyme is transported to the endoplasmic reticulum cavity by transporter associated with antigen processing (TAP), and then combined with major histocompatibility complex I (MHC I) to further present to the cell surface, then CD80+ Cytotoxic lymphocyte (CTL) was activated for specific identification of cull 50. The TAP of is located on the endoplasmic reticulum and cis-Golgi membrane in this process. TAP is composed of two subunits TAP1 and TAP2, and the loss of any subunit will make TAP ineffective and lead to the significant down-regulation of MHC I on the cell surface 50. Lin, T.C., et al. found that EBV miR-BHRF1-3 and EBV miR-BART17 directly target the 3'UTR of TAP2 mRNA, weaken EBV antigen presentation and MHC I cell surface expression, thereby inhibiting CD80+CTL cells' role in endogenous antigen recognition and killing host cells, resulting in immune escape 51.

When the host EBV antigen is presented to the cell surface, it not only activates the CTL to kill the cells but also activates antigen-presenting cells (APC) to uptake and process the antigen. In this process, the exogenous EBV antigen passes through the lymphocyte antigen (Lymphocyte antigen, LY) transports from the cell surface to lysosomes or endosomes, degraded to antigenic peptides by lysosomal enzymes or endosomal enzymes, and combined with major histocompatibility complex II (MHC II) 52. Lymphocyte antigen 75 (LY75) plays an important role in the transfer of exogenous EBV antigen from the cell surface to lysosomes or endosomes and activates CD4+ helper T cell (Th cells) and CD8+ CTL Cells 53. Skalsky, R.L., et al. found that EBV miR-BART1-5p can directly target LY75. Overexpression of EBV miR-BART1-5p in EBV-associated tumor cells can inhibit the expression of LY75, and promote tumor cells to get rid of the recognition and monitoring of CD4+ Th cells and CD80+ CTL cells 54.

After EBV antigen is transported from the cell surface to the lysosome or endosomes, it needs to be degraded into an antigenic peptide by the lysosomal enzyme or endosomal enzyme, and then the processed antigen is presented to CD4+ Th cell to activate the immune response by combining with MHC II 55. EBV miRNAs can regulate the expression of various lysosomal enzymes, inhibit the degradation of antigens, block the binding of antigens to MHC II, and attenuate the activation of CD4+Th cells. It was found that the 3'UTR of mRNA of the three lysosomal enzymes IFI30, LGMN and CTSB in APC cells were targeted by EBV miR-BART1, EBV miR-BART2, and EBV miR-BHRF1-2, thereby inhibiting antigen processing, down-regulating the binding and presentation of EBV antigen peptide fragment and MHC II, and weakening the activation of CD4+Th cells, suppressing the immune response and assisting EBV immune escape 56. In addition, in addition to directly regulating antigen presentation, EBV miRNAs can indirectly inhibit the expression of costimulatory molecules and MHCs to block antigen presentation, inhibit subsequent immune activation, and evade the monitoring of host immune system 43, 56.

## EBV miRNAs regulate the activation of immune cells

After EBV infects the host, it does not initiate lytic replication immediately but is in a state of persistent latency. When the host's immunity declines, the EBV in the latency state can be activated immediately, forming a recurrent infection, and a large number of synthetic EBVs self-products, including immune antigens such as EBNAs and LMPs, cause host immune responses. First, activate humoral immunity, induce the production of anti-EBNA antibodies, anti-EA antibodies, anti-MA antibodies and anti-VCA antibodies to resist EBV invasion, but that does not completely eliminate the invading virus, especially for latent infection of EBV. Cellular immunity plays an important role in monitoring viral activation and clearance of host cells at this time 57, 58. In the process of cellular immune response, the two most important roles are lymphocytes and host cells. Studies have found that EBV miRNAs can not only regulate the expression and presentation of EBV antigen in host cells but also regulate the activation and toxicity of immune cells and assist host cells in immune escape in the process of host immune defense.

### Direct regulation of immune cell activation

When the EBV infects the host, the host will produce a ligand-protein MICB, MICB is a ligand for the NKG2D receptor, and the NKG2D receptor is a receptor on the NK cell. Its activation can effectively activate NK cells, by recognizing MICB ligands on the host, and can eliminate infected host cells. Nachmani, D., et al. found that EBV-miR-BART2-5p inhibits the expression of MICB in the host cell by targeting the MICB mRNA on the host cell, reduce the sensitivity of identifying of the host cell by NK cells, block the activation of NK cells, thus protecting the host cell, escape immune recognition and killing of NK cells 59.

When EBV re-infect the host, dormant B cells, driven by the germinal center, can rapidly differentiate into memory B cells. This process is critical for removing the EBV of the intrusion. EBF1 is a transcription factor of B lymphocytes, and is a necessary condition for the development of germinal centers 60. And EBF1 also targets many B lymphocyte-specific genes, including Paired box 5(PAX5) and B cell receptor (BCR), these two genes are essential for maintaining the function of mature B lymphocyte^61^. Ross, N.et al. found that EBV-miR-BART11-5p can binds to the 3'UTR of EBF1 mRNA, inhibits the expression of EBF1, and negatively regulates the proliferation and differentiation of B lymphocytes, thereby blocking the rapid differentiation and immune activation of B lymphocytes 62.

The rapid differentiation of B lymphocytes in dormancy does not only require germinal center drive, but BCR and PRDM1 are equally important for antigen recognition, antigen-stimulating signal transduction, B lymphocyte activation and rapid differentiation 63, 64. Chen, Y., et al. found that EBV miR-BHRF1-2-5p and EBV miR-BART2-5p in EBV positive B lymphocytes can regulate the BCR pathway, affect the transformation of B cells with latent EBV infection during the Latency and lysis period, inhibit the reactivation and differentiation of B lymphocytes, and assist the immune escape of infected host cells 65. PRDM1 is the main regulator of the terminal differentiation of B lymphocytes and a tumor suppressor gene. Ma, J., et al. have found that PRDM1 expression is very low in EBV+ diffuse large b-cell lymphoma (DLBCL) and Hodgkin's lymphoma, while inhibiting the expression of endogenous EBV miR-BHRF1-2 can significantly improve the expression of PRDM1. Further studies have found that EBV miR-BHRF1-2 can targets and down-regulates PRDM1 expression and inhibits B lymphocyte differentiation and immune activation by targeting the 3'UTR' of PRDM1 mRNA, at the same time promote the B lymphocyte differentiation and B lymphocyte immortalized 66.

After EBV infection of lymphocytes, EBV miRNAs can reduce the sensitivity of lymphocyte immune recognition, reduce the cytotoxicity of lymphocytes, protect host cells, and evade immune attack. IPO7 is a nuclear receptor protein and a transcription factor that regulates immune tolerance. The expression level of IPO7 is positively correlated with the cytotoxicity of T lymphocytes 67. Dolken, L., et al. found that in EBV-positive T lymphocytes, EBV-miR-BART3 targets the 3'UTR of IPO7 mRNA, inhibits the expression of IPO7, reduces the cytotoxicity of T lymphocytes, and assists host cells in immune escape 68. In addition, Mucosa-associated lymphoid tissue lymphoma transport protein 1 (MALT1) plays an important role in the activation of CD4+ T cells in the immune response 69, while EBV miR-BHRF1-2-5p inhibits the expression of MALT1, thereby inhibiting the activation and cytotoxicity of CD4+ T cells 70, 71.

### Indirect regulation of immune cell activation

#### Interleukin expression and release

After EBV infection in the host, the expressed EBV antigen is exposed by endogenous and exogenous presentation, recognized by lymphocytes, and activated by the immune response of related lymphocytes to eliminate EBV. Interleukin (IL) mediation during this process is necessary for lymphocyte activation, proliferation and differentiation, and activation of further inflammatory responses 72. EBV miRNAs can target the inhibition of IL or IL receptor expression, block IL-mediated lymphocyte differentiation and immune activation. For example, IL1R1 is a receptor for IL-1. When EBV virus invades, IL-1 signaling pathway plays an important role in triggering an inflammatory response and host innate immune response 73. Skinner, C.M., et al. that EBV miR-BHRF2-5p can directly target The IL-1 receptor (L1R1), the activation of IL-Lβ-induced NF-kB, and inhibits activation of host lymphocytes and immune responses 74. IL-6 can promote the proliferation of B cells and T cells, stimulate the activation of CTL cells, and participate in the inflammatory response 75, while EBV miR-BART6-3p can target IL-6 receptors and inhibit lymphocyte immune activation 76. IL-12 promotes T cell proliferation, induces Th0 cells differentiation into Th1 cells, and activates cytotoxicity of CTL cells and NK cells 77, while EBV miR-BART1, EBV miR-BART2, EBV miR-BART22 and EBV miR-BHRF1-2 significantly inhibited the expression and release of IL-12 56. Similarly, EBV miR-BART15-3p directly targets the 3'UTR of inflammasome NLRP3 mRNA, inhibits NLRP3 expression, and indirectly inhibits IL-1β expression, thereby attenuating host-induced immune responses 78.

#### Interferon expression and release

When the host is infected by EBV, it not only expresses and releases IL to resist virus infection, but also expresses Interferons (IFNs) and releases. IFNs can up-regulate the activity of various immune cells, such as macrophages, dendritic cells (DC), B lymphocytes, T lymphocytes and NK cells, so as to exert anti-viral and anti-tumor effects 79. EBV-miRNAs were found to target and regulate the genes involved in the IFNs signaling pathway, including T-bet, CXCL11, CLEC2D, RIG-I, and CREBBP, to inhibit the expression and release of INFs, and inhibiting the activation of immune responses and immunotoxicity.

The T cell-mediated transcriptional regulator T-bet is a transcriptional activator of IFN-γ and regulates the production of IL-2 and Th2 cytokines 80. Tokunaga, R., et al. has found that EBV miR-BART20-5p directly targets the inhibition of T-bet expression in invasive EBV+ nasal NK/T-cell lymphoma, and EBV miR-BART8 can indirectly block IFN-γ/STAT1 pathway, can inhibit IFN-γ activation of the immune response. CXCL11 is an interferon-induced chemokine, and its corresponding chemokine receptor CXCR3 is expressed on the surface of NK cells and Th1 cells. When EBV invades host cells, CXCL11 on the host cells can specifically bind to CXCR3, thereby recruiting NK Cells and Th1 cells, and activate them 81. Xia, T et al. predicted that EBV miR-BHRF1-3 directly targets CXCL11, and inhibits the expression of endogenous EBV miR-BHRF1-3 in EBV-positive B cells, which can significantly increase CXCL11 mRNA levels, indicating EBV miR-BHRF1-3 directly or indirectly regulate CXCL11 expression, inhibit the recruitment and activation of NK cells and Th1 cells 82, 83. CLEC2D is expressed on the surface of B lymphocytes, promotes the expression and release of IFN-γ by interacting with CD161 molecules on lymphocytes, and activates NK cells and T cells 84, while EBV miR-BART1-3p and EBV miR-BART3-3p can target the 3'UTR of CLEC2D mRNA, inhibit the expression and release of IFN-γ, and block the activation of NK cells and T cells 54. The retinoic acid-inducible gene-I (RIG-I) recognizes EBV nucleic acid in the cytoplasm and induces the expression and secretion of IFNs, activates NK cells and T cells 85, while EBV miR-6-3p can bind to the 3'UTR of RIG-I mRNA inhibits the expression and release of IFNs, and inhibits the immune activation of lymphocytes 86. The CREBBP gene is a coactivator of transcription of the type I IFN signaling pathway, and activates the expression and release of IFNs 87. It was found that EBV-miR-BART16 can target CREBBP and block the expression and release of virus-induced type I IFNs, suppress immune activation and response and assist host immune escape 88, 89.

#### Exosomes assist tumor cell immune escape

In addition to directly acting on genes in host cells, EBV-miRNAs synthesized by EBV can also secrete exosomes through host cells, and use exosomes as vectors to transfer EBV-miRNAs from host cells to adjacent or distant cells. It regulates the expression of genes in other cells, exerts biological functions, and cooperates to achieve immune evasion 90. It has found that in EBV-related tumors (such as nasopharyngeal carcinoma, Burkitt's lymphoma, Hodgkin's lymphoma, NK/T-cell lymphoma, gastric cancer), exosomes carrying EBV-miRNAs can promote tumor cell immune escape and promote progression of the tumor 91. In EBV-associated tumor tissues, tumor infiltrating lymphocytes (TILs) can inhale EBV-miR-BARTs by endocytosis, and these EBV miRNAs can significantly reduce the immune recognition and cytotoxicity of TILs. For example, EBV-miR-BART3 inhaled can target the 3'UTR of IPO7 mRNA in TILs, inhibit the expression of IPO7 in TILs, and reduce its specific cytotoxicity against tumor cells, thus assisting tumor cell immune escape 68.

In general, EBV-miRNAs are delivered to immune-related cells by exocytosis release, transmission and endocytosis, regulating the expression of related genes, suppressing immune responses, synergistically promoting the immune escape of the host, and promoting tumor development.

## Conclusions

To sum up, after EBV infection host cells, in the face of the host in the process of the body's immune response of attack, EBV miRNAs by adjusting the EBV antigen expression in host cells, interfere with antigen presentation, regulating effect and toxicity of lymphocyte, so as to derive a series of getting rid of host immune surveillance and immune attack strategy, to assist the host cell and EBV immune escape, achieve long-term latent EBV infection, and promote the development of host cells to immortalization and tumors. And also can use host secrete exosomes, EBV miRNAs are transferred to adjacent or distant tumor cells and play a corresponding biological function to assist the immune escape of tumor cells.

At present, there are still many biological functions and mechanisms of EBV miRNAs that are not well clear. Even the well-defined functional EBV miRNAs may have other biological functions and mechanisms that have not yet been discovered, and there is still much work to be done. More or comprehensive disclosure of the targets and mechanisms of EBV miRNAs will play an important role in elucidating the mechanisms between EBV and the host immune system, EBV and host cell immortalization. It may provide an alternative immunotherapy approach for EBV-associated tumors as a potential target.

## Figures and Tables

**Figure 1:**
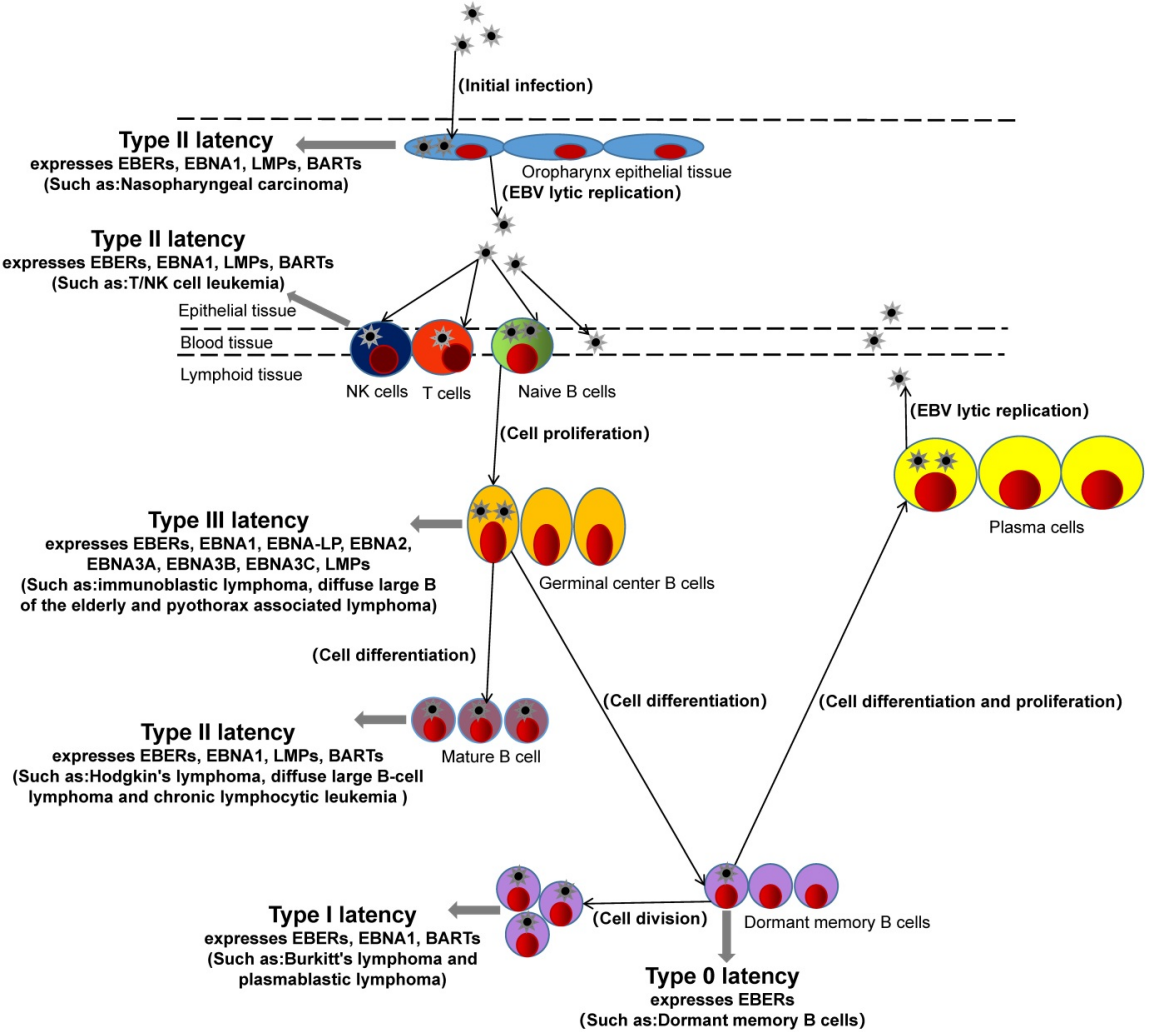
The formation of EBV latent infection

**Figure 2:**
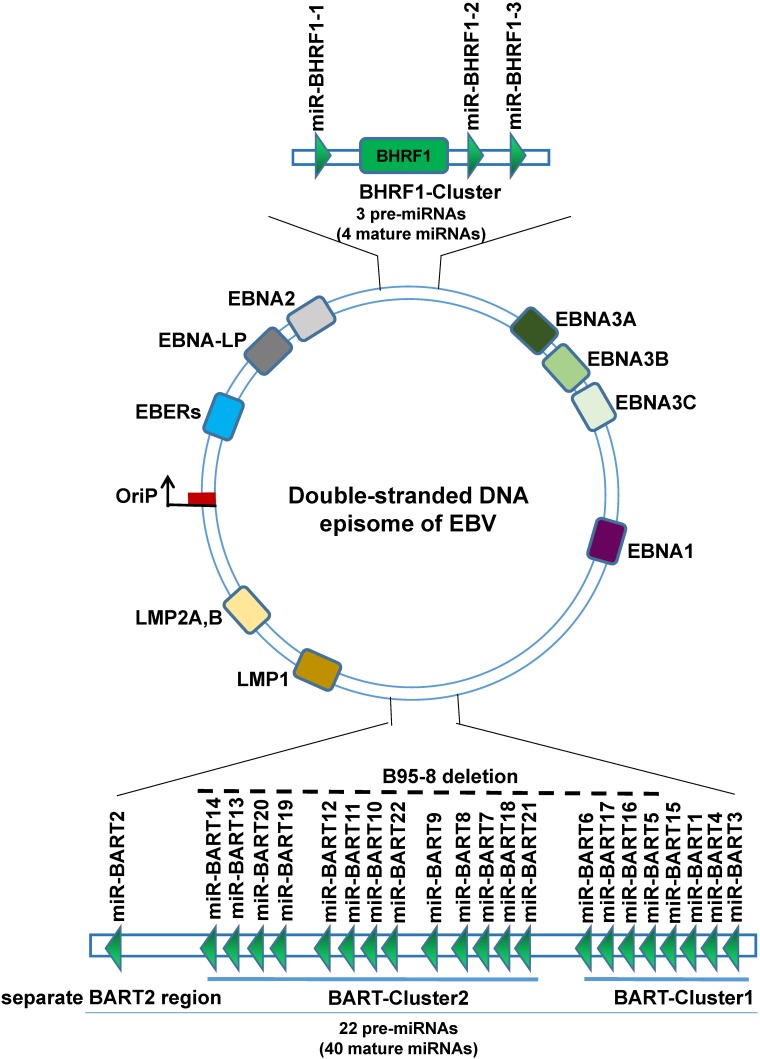
Schematic representation of the genomic locations of EBV-encoded miRNAs. The relative positions of EBV latent and EBV encodes 25 pre-miRNAs genes on the genome are indicated.

**Figure 3:**
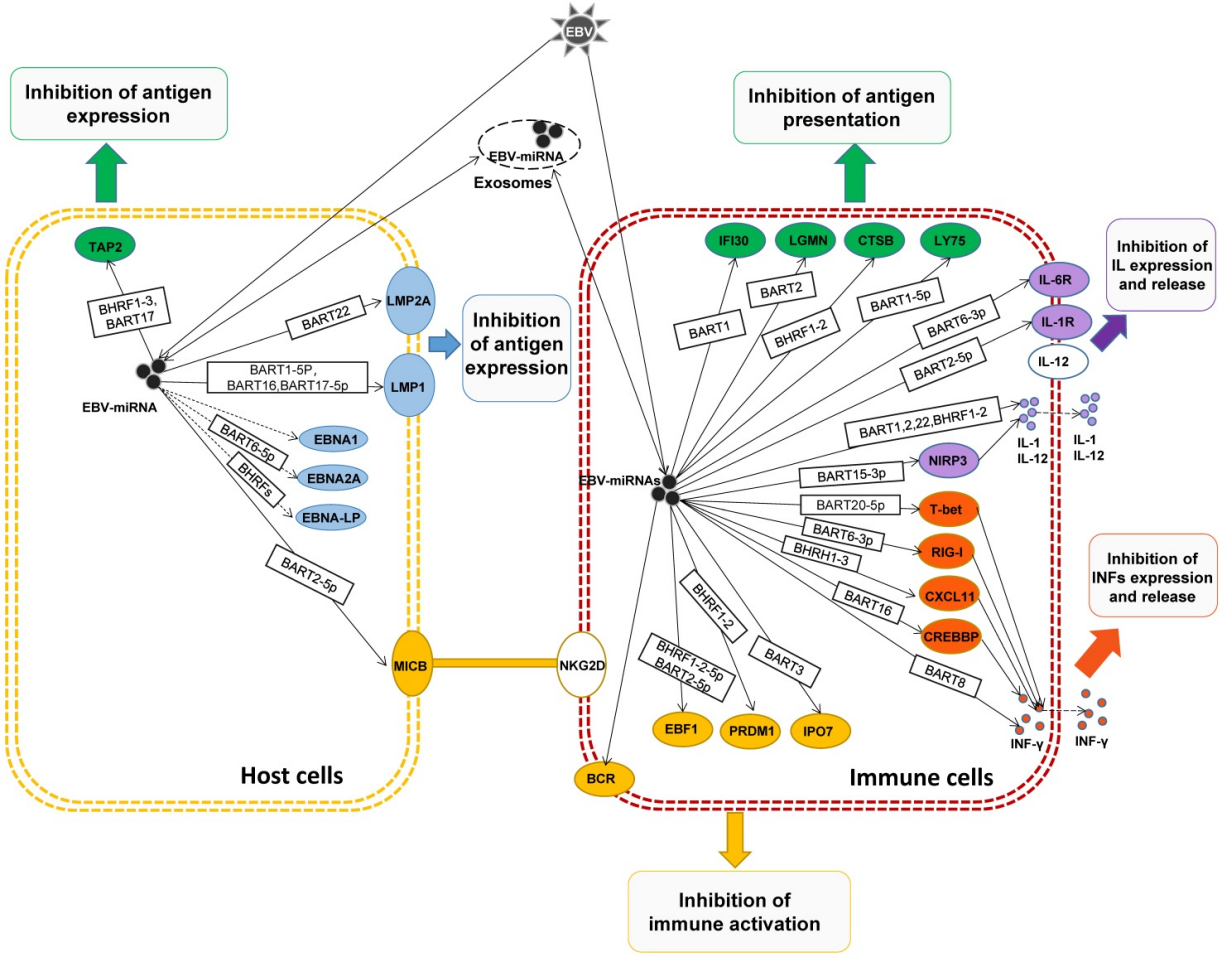
EBV miRNA regulate host immune responses
